# Case Report: Successful treatment of severe pneumocystis carinii pneumonia in a case series of primary nephrotic syndrome after receiving anti-CD20 monoclonal antibody therapy

**DOI:** 10.3389/fped.2022.1067634

**Published:** 2023-01-04

**Authors:** Lili Liu, Weihua Zheng, Ping Wang, Ying Wu, Guanghua Zhu, Rong Yang, Li Gu, Wenyan Huang, Yulin Kang

**Affiliations:** ^1^ Department of Nephrology and Rheumatology, Shanghai Children’s Hospital, School of Medicine, Shanghai Jiao Tong University, Shanghai, China; ^2^ Department of Pediatrics, Shanghai Tenth People’s Hospital, School of Medicine, Tongji University, Shanghai, China

**Keywords:** pneumocystis carinii pneumonia, primary nephrotic syndrome, rituximab, metagenomic next-generation sequencing, pediatrics

## Abstract

Rituximab is emerging as a new steroid sparing agent in children with difficult-to-treat nephrotic syndrome due to its ability of depleting CD20-positive B cells. Life-threatening adverse events such as pneumocystis carinii pneumonia may occur even though it seems to be well tolerated. Since rituximab is wildly used in immune-mediated diseases, it is important to manage its severe adverse events. To explore the importance of early diagnosis and treatment of pneumocystis carinii pneumonia in children with primary nephrotic syndrome (PNS) after receiving rituximab therapy, we retrospectively analyzed the clinical data of PNS patients younger than 18 years old with pneumocystis carinii pneumonia who were hospitalized in our center. Clinical features and laboratory test results were retrieved from the electronic medical records. Severe pneumocystis carinii pneumonia occurred in one child with steroid resistant nephrotic syndrome and two with steroid dependent nephrotic syndrome patients after rituximab treatment. These patients were diagnosed in time by metagenomic next-generation sequencing (mNGS) for pathogen detection. Fortunately, all three patients survived after antifungal treatment and achieved complete remission eventually. In conclusion, early diagnosis by using mNGS and timely antifungal treatment is the key to successful management of pneumocystis carinii pneumonia in children with difficult-to-treat PNS.

## Introduction

Primary nephrotic syndrome (PNS) is characterized with heavy proteinuria, hypoalbuminemia, hyperlipidemia and edema in children. It may progress to end stage renal disease if proteinuria persists (1). Glucocorticoid is the first line therapy in patients with PNS. If patients develop steroid dependence, immunosuppressants including cyclophosphamide, cyclosporine A, tacrolimus, cyclosporine and mycophenolate were often used. The prognosis has been greatly improved since immunosuppression therapy was introduced (2, 3). However, adverse events such as obesity and infection were widely reported after long-term steroid and immunosuppression therapy. Thereafter, monoclonal antibodies were developed for these immune-meditated diseases due to its higher efficacy and less side effects.

Rituximab is a chimeric monoclonal antibody that induces peripheral CD20 positive B cell depletion. It emerges as a new steroid sparing agent in children with refractory nephrotic syndrome (4). It has been shown that rituximab induces remission and reduces steroid exposure in patients with steroid dependent nephrotic syndrome (SDNS) (5–7). In addition, rituximab has also satisfactory therapeutic effects in patients with steroid resistant nephrotic syndrome (SRNS) (8). Generally, rituximab is a safe medication for children with PNS. However, rituximab also lead to pneumocystis carinii pneumonia (PCP) which is a rare but serious complication with diffuse grid-like shadows in the computerized tomography (CT) of lungs (9). High mortality was reported in patients with PCP (10). Thus, it is necessary to enhance our understanding of this severe infection.

PCP, also known as pneumocystis jirovecii pneumonia (PJP), is an opportunistic fungal infection that often occurs in immunocompromised population, most commonly seen in patients with acquired immunodeficiency syndrome (AIDS) (11). Although it is a major cause of death in patients with HIV, the advent of effective therapeutic and prevention strategies has led to a significant decline in its incidence. However, it is also a fatal pneumonia in non-HIV immunocompromised patients (12). Furthermore, patients with PCP who are not HIV-infected usually exhibit more severe and higher mortality than HIV-infected patients (13). The use of glucocorticoids and immunosuppressants is the risk factor for non-HIV patients with PCP (14, 15). As a novel monoclonal antibody, rituximab is increasingly used in patients with difficult-to-treat PNS (16, 17). Therefore, the awareness of life-threatening adverse events should be raised after rituximab treatment.

Here we reported three cases of severe PCP in children with difficult-to-treat PNS after rituximab treatment. It highlighted the importance of early diagnosis and timely antifungal treatment in the management of non-HIV immunocompromised PCP patients.

## Case series

### Case 1

**Figure 1:**
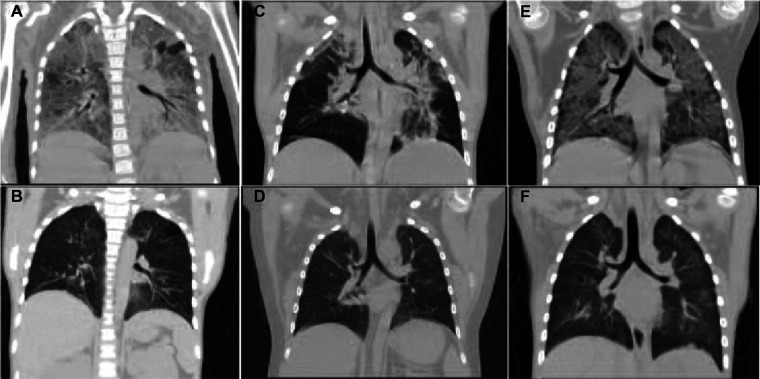
The CT images of lungs in three PNS cases with PCP. It showed that extensive exudation in both lungs was improved significantly after antifungal treatment. (**A,B**) The CT images of lungs before and after anti-fungal treatment in Case 1. (**C,D**) The CT images of the lungs before and after treatment in Case 2. (**E,F**) The CT images of lungs before and after treatment in Case 3.

A 7 years old girl with facial edema and oliguria was diagnosed with PNS in our hospital. After 2 weeks of prednisone treatment, serum creatinine (112 mmol/L) and 24 h urinary protein (3,000 mg/m^2^) did not improve. Thus, patient received renal biopsy which revealed that it was minimal change nephrotic syndrome (MCNS). Subsequently, this patient received 3 consecutive days of intravenous methylprednisolone (20 mg/kg of body weight per day) pulse therapy followed by oral prednisone treatment (2 mg/kg/day) for 6 weeks and 4 times of intravenous cyclophosphamide (0.5 g/m^2^ biweekly) pulse therapy. However, complete remission was still not achieved. According to the 2021 KIDGO guidelines, the diagnosis of SRNS and acute kidney injury (AKI) at the stage 2 was established eventually. However, there were no gene mutations. For alleviating proteinuria, a single dose of rituximab (375 mg/m^2^) was given intravenously. Three days later, the number of CD20 positive B cell decreased to zero. Ten days later, she presented with cough, difficult breathing and fever. The chest CT showed that ground-glass opacities in both lungs (Figure 1A). Moreover, serum creatinine increased to 162 μmol/L. Pneumocystis carinii was found in sputum at the early stage by using metagenomic next-generation sequencing (mNGS) which emerges as a promising method for universal pathogens identification in the specimen due to its properties of simultaneous identification and genomic characterization of bacteria, fugus, virus and parasites (18). With severe respiratory failure, she was supported with mechanical ventilation. Since renal function deteriorated, continuous renal replacement therapy (CRRT) was performed. After 24 days of intravenous caspofungin and sulfamethoxazole-trimethoprim (SMZ-TMP) treatment, the symptoms of lung injury was improved. The chest CT showed obvious improvement of pulmonary inflammation (Figure 1B). Surprisingly, the child became sensitive to steroid and achieved complete remission in the 15 months of follow up. The percentage of CD20 positive B cells were 2.0%, 5.2%, 12.1%, 16.2% in the 3rd, 6th and 12th month respectively.

### Case 2

A 5-year-old boy with steroid dependent MCNS was admitted to our hospital. He has been treated with prednisone, cyclosporine and 9 consecutive intravenous pulses of cyclophosphamide (0.5 g/m^2^ monthly) in the past 3 years. However, the patient still relapsed frequently. Therefore, a single dose of rituximab (375 mg/m^2^) was given intravenously in the latest episode of relapse. Three days later, the number of CD20 positive B cell decreased to be 0.19%. The urine protein turned to be negative within 1 week. Unfortunately, the patient presented with high fever (39°C) and cough on Day 13 after rituximab treatment. The complete blood count (CBC) test showed that leukocytes 4.76 × 10^9^/L, hemoglobin 136.0 g/L, platelets 333.0 × 10^9^/L, neutrophil percentage 91%, C-reactive protein 6 mg/L. Serum albumin 36.65 g/L, globulin 18 g/L, sodium 140 g/L, potassium 4.5 g/L, calcium 2.06 g/L. Liver and renal function was normal. Urinalysis: urinary protein ++. The chest CT showed ground-glass opacities in both lungs (Figure 1C). mNGS showed there was pneumocystis carinii in bronchoalveolar lavage fluid. However, it was negative in sputum culture at the same time. Anti-infective treatment with meropenem, amoxicillin clavulanate potassium, oral SMZ-TMP and caspofungin were given. Meanwhile, the patient received mechanical ventilation due to severe respiratory distress. After 20 days of the treatment, dyspnea disappeared and the sign of lung infection was greatly improved (Figure 1D). Two months after RTX treatment, prednisone was terminated. One week after anti-PCP treatment, urinary protein turned to be negative. Moreover, the patient achieved complete remission in the following 24 months. The percentage of CD20 positive B cells were 2.6%, 5.9%, 16.1%, 24.2% in the 3rd, 6th, 12th and 24th month respectively.

### Case 3

A 3-year-old boy with PNS was transferred to our hospital because of edema, proteinuria, hypoalbuminemia and hyperlipidemia. Initially, he responded well to steroid treatment. However, SDNS was developed eventually. The renal biopsy was performed 11 months after the onset of the disease. MCNS was the renal pathological change. Therefore, he was treated with a single dose of RTX (375 mg/m^2^). Three days later, the number of CD19 positive B cell decreased to be less than 0.5%. Complete remission was achieved in 1 week. However, he presented with cough and shortness of breath 6 weeks after RTX treatment. CBC test: leukocytes 8.83 × 10^9^/L, hemoglobin 138 g/L, platelets 465 × 10^9^/L, neutrophil percentage 64.2%, C-reactive protein ≤5 mg/L. Blood biochemical test: alanine aminotransferase 15 U/L, aspartate aminotransferase 22 U/L, total protein 52.37 g/L, albumin 26.67 g/L, serum creatinine 36 ummol/L. The 24 h urinary protein was 1100.0 mg/m^2^. The chest CT showed diffuse exudation with interstitial changes in both lungs (Figure 1E). mNGS of bronchoalveolar lavage fluid revealed pneumocystis infection. He received high-flow of oxygen therapy. After 4 weeks of oral SMZ-TMP, caspofungin, meropenem and minocycline treatment, the symptoms of lung infection disappeared and complete resolution of inflammation was found in chest CT images (Figure 1F). Twenty-four hour urinary protein turned to be normal. Moreover, the patient achieved complete remission in the following 12 months. The percentage of CD20 positive B cells were 3.5%, 6.3%, 17.8% in the 3rd, 6th and 12th month respectively.

## Discussion

Patients with nephrotic syndrome may develop PCP after steroids and immunosuppressants treatment. Particularly, PCP may occur under the condition of secondary immunodeficiency caused by rituximab therapy (16, 17). It is a big challenge to decrease the high mortality of PCP in immunocompromised patients. Recently, severe PCP in three children with PNS after rituximab treatment was successfully treated in our center.

PCP is an opportunistic respiratory infection that often occurs in immunocompromised populations (19). Pneumocystis infection may be asymptomatic in immunocompetent patients, but mortality is high in immunocompromised patients (20). The risk of pneumocystis infection is significantly higher when the number of CD4+ T-cell is below 200/mm^3^ (21). PCP is not only prevalent in patients with AIDS, but also occurs in kidney diseases, lymphoma, Wegener's granulomatosis, pemphigus, granulomatosis with polyangiitis and rheumatoid arthritis (22–27). Patients with SDNS or SRNS were commonly treated with immunosuppressants including rituximab in an attempt to induce remission of the disease. These three patients hospitalized in our center became steroid resistance or dependent after standard steroid therapy. They developed severe PCP after being in an immunodeficient state secondary to rituximab treatment. Fortunately, all three patients recovered completely after antifungal treatment. Therefore, it is necessary to increase the awareness of PCP in immunocompromised patients.

In immunodeficient patients, PCP is a life-threatening disease. Typical PCP is featured by dyspnea, fever and cough. The CT scanning of lungs shows diffuse ground-glass opacities. However, the early clinical presentation is atypical in most patients. Pneumocystis jirovecii is a fungus that is extremely difficult to culture *in vitro*. The diagnosis of PCP is currently based on examination of respiratory samples, including microbiological tests (e.g., polymerase chain reaction, microscopy, β-d-glucan) and radiological methods (28). Since clinical manifestations of PCP vary widely among patients, the diagnosis should not only rely on clinical manifestations and radiological findings, but also the laboratory results of pneumocystis infection (29). Serology and microscopic tests of respiratory samples is feasible, but they are limited to false positive and low sensitivity. Currently, polymerase chain reaction is widely used in the diagnosis of PCP, but it requires pathogen hypothesis in advance and is also limited to the low specificity (30). Thus, the key of early diagnosis is to have an assay which could detect expected and unexpected pathogens with high sensitivity and specificity.

mNGS technology is increasingly used for the detection of pathogenic microorganisms by detailed and unbiased analysis of their DNA or RNA content (30, 31). Thus, it is capable of simultaneously detecting pathogens such as bacteria, virus, fugus and parasites from clinical samples. Not only mNGS is fast, unbiased and highly sensitive, but also it can identify pathogens that are not identified by conventional diagnostic methods (32). This technique has been widely used for the detection of serious lung infections (33, 34). It has been known that mNGS has higher positive rates than the conventional tests for pathogens identification in immunocompromised and immunocompetent patients. Moreover, it is capable of detecting mixed pathogens which are more common in immunocompromised patients (35, 36). Blood and alveolar lavage fluid are the most commonly used samples for mNGS detection. It has been shown that pathogenic microorganisms detected by mNGS in alveolar lavage fluid and blood samples achieve a perfect match (37). In our three cases, mNGS of respiratory samples is the key for early diagnosis of PCP. Therefore, mNGS is emerging as a promising technique for the diagnosis of PCP.

The treatment of PCP is also full of challenges. SMZ-TMP is the first-line agent for treatment and prevention of PCP (19). It has good tolerability, even though it may have some side effects with long-term use. In Case 1, intravenous SMZ was used for keeping the stable blood concentration, as CRRT could remove it. Therefore, early diagnosis and timely treatment of PCP are crucial for improving the prognosis. Furthermore, our data showed complete remission was achieved in patients with SRNS and SDNS after the recovery of PCP infection. It remains unclear whether long-term complete remission in these 3 cases was attributed to the effectiveness of Rituximab or (and) PCP infection, as it has been shown that a dramatic decrease in immunosuppression may lead to immune reconstitution (38). Pneumocystis-associated immune reconstitution of the immune response occurs in AIDS patients with PCP (39). Thus, the mechanism by which the primary disease appears to remit after pneumocystis infection in immunocompromised patients needs to be investigated in future. Additionally, long-term follow-up is important for the prevention and treatment of this rare complication.

## Limitations

This study also has some limitations. Although mNGS facilitates early diagnosis of PCP, the high costs limits its widespread clinical applications. Moreover, the association between PCP and long-term complete remission in children with PNS needs to be verified in future.

## Conclusion

PCP is a life-threatening disease which often occurs in immunocompromised patients. Early diagnosis by using mNGS and timely antifungal treatment is the key to successful treatment of PCP infection in children with PNS.

## Data Availability

The raw data supporting the conclusions of this article will be made available by the authors, without undue reservation.
